# Dismemberment and Body Encasement—Case Report and an Empiric Study

**DOI:** 10.3390/biology11020328

**Published:** 2022-02-18

**Authors:** Jana Matzen, Benjamin Ondruschka, Antonia Fitzek, Klaus Püschel, Eilin Jopp-van Well

**Affiliations:** Institute of Legal Medicine, University Medical Center Hamburg-Eppendorf, Butenfeld 34, D-22529 Hamburg, Germany; b.ondruschka@uke.de (B.O.); a.fitzek@uke.de (A.F.); pueschel@uke.de (K.P.); eilin.jopp@web.de (E.J.-v.W.)

**Keywords:** dismemberment, postmortem CT, X-ray, forensic anthropology, encasement

## Abstract

**Simple Summary:**

We report a rare case of dismemberment and encasement from our catchment area. Various body parts were encased in concrete and thrown into a small stream behind the perpetrators’ farm and visualized with postmortem computed tomography (pmCT) and mobile X-ray device. For reconstruction purposes the head and paw of a pig were encased in concrete in a comparable manner to an actual victim and regularly examined with imaging techniques, to figure out the value of such efforts in cases where concrete is used and to understand changes in the animal’s body over time.

**Abstract:**

The mutilation and encasement of corpses are rare in daily forensic work, but when they occur, close cooperation between different disciplines, such as legal medicine and forensic anthropology, is necessary to obtain the most valuable results. One forensic examination method is the radiological evaluation of victims or body parts by postmortem CT (pmCT) and X-ray images. In relation to a case described in this paper, an empirical study was conducted to figure out the value of radiological imaging and the ability to visualize and temporally classify changes in a corpse encased in concrete. For this purpose, the head and paw of a pig were encased in concrete and scanned regularly over a period of one year. Body parts such as the head and paw are clearly visible on X-ray images. Although decay-related changes are shown, a specific minimum time interval cannot yet be found, as these changes occur continuously in lesser amounts.

## 1. Introduction

Cases of encasement and dismemberment are rarely seen in our catchment area in North Germany and, therefore, pose significant local challenges. When they do occur, their circumstances and dynamics are of great interest.

In the forensic context, body disposal, or “dumping” describes the process of disposing of a corpse. According to the literature, the most common method of disposal is dumping the body in water [1], while bricking and encasing are among the rarer forms [2]. According to Schneikert, there are different degrees of immersion, from the first, and least-invasive to the third and most-invasive degree. Encasement and dismemberment both fall into the third category [3]. A distinction is also made between direct and indirect dumping. Direct dumping involves destroying characteristic features that could assist in identification, while indirect dumping involves hiding the body without destroying possible identifying evidence [1].

Dismemberment can be divided into defensive and offensive types. Defensive dismemberment is characterized by a systematic approach, which serves the purpose of hiding the body. Offensive dismemberment, on the other hand, is often characterized by an indiscriminate approach and the mutilation of the genitals [4,5]. However, there are mixed forms between both variants.

Dismemberment cases tend to be underreported [6,7]. The fascination with such cases is exemplified in many studies and case reports in the literature [5,8,9,10,11,12,13,14,15]. Black et al. [15] and Maiese et al. [10] further emphasize the importance of a multidisciplinary approach to dismemberment cases, involving forensic anthropologists, radiologists and odontologists, among others. Over the last two decades, seventeen different cases of criminal dismemberment have been investigated at the Institute of Legal Medicine at the University Medical Center Hamburg-Eppendorf in Germany. Cases of encasement are even rarer: between 2000 and 2020, only three cases were found in Hamburg (own data). The combination of a dismemberment and encasement occurred only once—in the case described in this article. The combination of encasement and dismemberment was also rarely found in the literature. This special combination was mentioned in the case reviews by Dedouit et al. [16], and in several cases by Toms et al. [17].

A study by Toms et al. [17] examined cases of encapsulated dumping (*n* = 18) in Los Angeles. Investigators used both metal detectors and radiological equipment to find the body or body parts inside the concrete. Specific to these cases, the authors reported difficulties in estimating the postmortem interval (PMI), but also the positive effects of concrete in terms of preserving the body for longer and the possibility of making negative casts of, for example, the hands and face, thus aiding the identification process.

An article by Dressler and Madea [18] describes the case of a shot victim, who had been immured in a wall in the basement of his house. The body was found 8 months afterwards, but forensic pathologic investigations were still possible due to the cold environment and resulting delay in putrefactive changes.

In another study in Germany, the authors described six cases where corpses were encased in concrete and/or bricks. The results of these cases also showed slowed decomposition compared to non-sheathed cases, and therefore difficulties in an uncritical estimate of PMI. These victims were all immured or concreted into floors or walls; therefore, only discovered after confessions of the perpetrators or police investigations. However, no radiological evidence was used in any of the cases [19].

Dedouit et al. [16] used CT to analyze human bone in a concrete block. They concluded that CT has great potential to distinguish bones from cement before removal from concrete to prevent the generation of preparation artefacts and to obtain data that might be lost during removal from concrete.

Aside from concrete, quicklime is another material often used to disguise the victim. In the case described, it was also used for the torso. The literature search revealed two studies on the effects of quicklime on buried corpses [20,21].

In crime scene investigations of dismembered bodies, there are technical modalities that can help specialists in the identification and analysis of body parts. Particularly in the case of entrapped victims, there are obvious difficulties in finding the victims in the first place, and then excavating them safely to preserve clues as much as possible.

A study by Ruffell et al. suggests a specific search strategy in trapped or walled-in victims, including, for example, geophysics, search dogs and the use of imaging such as X-rays, ultrasound, or gamma rays, followed by drilling and the use of an endoscope [22].

One possibility for cement blocks with unknown contents, as in our case, is a mobile X-ray device, e.g., the so-called ScanVan (Manufacturer: Smiths Heimann GmbH; Wiesbaden, Germany). This device is normally used to scan luggage at airports and national borders but can also identify and localize the contents of concrete blocks already at the site of discovery [23]. Another radiological modality is postmortem computed tomography (pmCT), which can be used for more detailed pictures in thin slices and potential three-dimensional reconstructions [2]; the remains are then transported to the responsible Institute of Legal Medicine or Department of Radiology.

### 1.1. Dismemberment Case Report

A few years ago, a man made a statement about the possible murder of a person who had been missing for two years. In this statement, an indoor riding arena was mentioned as a possible location of the body, which led to a police search at the victim’s property.

A human torso was found at the described location. During a later search for the remaining body parts, several concrete blocks were found in a stream behind the farm. As the specialists suspected that they might hold the missing parts, the blocks were scanned with a mobile X-ray machine of the Hamburg customs department.

**Figure 1:**
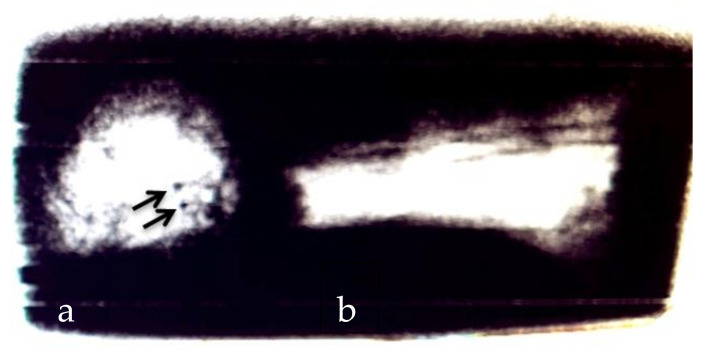
Position of the victim’s head (**a**) and thigh (**b**) in the concrete. Two roundish defects and possibly projectiles in the skull are visible and marked with arrows (picture provided by Hamburg customs department [23]).

With the help of this mobile X-ray device, the body parts could already be localized in the concrete on site (original image; Figure 1). Those X-ray images helped to carefully remove the remains with a percussion drill, hammer, and chisel. The remains were then taken to the Institute of Legal Medicine in Hamburg, where a pmCT and an autopsy of all body parts were carried out with subsequent DNA and toxicological examinations [23].

According to the autopsy report, the cause of death was a traumatic brain injury from two gunshots to the back of the head. The projectiles were both inside the skull. The body was dismembered into thirteen parts. Next to the torso, which was found in the indoor riding arena, were six concrete blocks holding the missing body parts. The body had been cut into feet, lower legs, upper legs, torso, upper arms, lower arms with hands and head. The cut wounds were in the lower third of the lower legs, the knee area, the neck of the femur, the elbow area, the head of the humerus and below the fourth cervical vertebra.

The penis and testicles were separated from the body and found together with other parts in another concrete blocks.

The skin of all body parts showed blackish-greyish discoloration, advanced putrefaction and adipocere formation. The torso also showed adhesions of soil and the presence of small maggots, while the encased body parts were completely free of insects. The brain tissue was softened but not completely decomposed, and the skull showed two roundish perforations, in the parietal region and just to the left of the occipital protuberance, with the gun shot angle running slightly upwards and horizontally, respectively.

The organized pattern and symmetrical approach to dismemberment suggests that this case falls into the category of defensive mutilation. This contrasts with the separation of the penis and testicles, which would be interpreted as a sign of offensive mutilation [4]. When questioning the perpetrator, however, any sexual motivation was denied, and the separation was explained with an accident. Due to this explanation, it ultimately remains unclear whether defensive and offensive dismemberment took place simultaneously in this case. Furthermore, this case would be classified as indirect dumping, as certain identifying features such as fingertips, teeth and facial features were still intact despite the dismemberment.

The weapons used in this case included a small caliber pistol for the murder and a knife, saw and axe for the dismemberment. The cut surfaces through the skin and soft tissues were smooth, indicating the use of a knife. The cut surfaces on the bones of the torso and the extremities were rough and had cracks and bone splinters, suggesting the use of an axe, while the bone margins of the head wounds had a smoother but undulated surface, suggesting the use of a saw.

Later, during the trial, the accused explained the sequence of events as follows: The victim was lured into a room of the house where he was shot twice in the head. Afterwards, the body was buried in the indoor riding arena, using quicklime to mask the smell. Later, the body was excavated, dismembered and most of the parts were put into several concrete blocks, while the torso was buried again.

### 1.2. Empirical Study

An empirical study was designed to develop a better understanding of the value, imageability and practical utility of radiological modalities such as X-ray and pmCT in comparable cases. The aim was to compare these two modalities and gain insights into their value and limitations in the special forensic circumstance of a combined dismemberment and encasement. The changes inside the encased corpse were documented over the period of one year to find out whether radiological equipment could be used, not only to identify and find human remains, but also to detect changes over time that supply information about the rate of decomposition inside the concrete.

## 2. Materials and Methods

For this empirical study, a pig’s head and paw from a local butcher were encased in concrete. These specific parts were chosen to provide some comparability with one of the concrete blocks in the real case, which contained a human head and one thigh. Since the exact nature of the concrete in the case was not known, commercially screed concrete was used (manufacturer: Bau Sys, Dortmund, Germany; solidity class C25/30, 40 kg to five liters of water). To illustrate gunshot wounds but not to hit the pig’s head, a projectile, provided by the colleagues from the Hamburg customs department was placed in an orbit. The pig was slaughtered for food on 8 September 2020 and dismembered afterwards using butcher blades. The head and paw provided, which were considered waste, were encased the next day.

**Figure 2:**
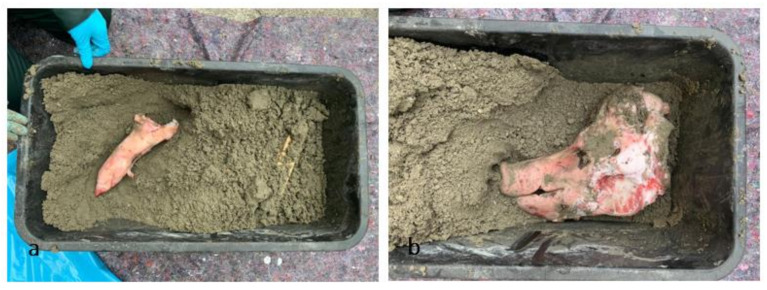
(**a**,**b**) Position of pig’s paw (**a**) and head (**b**) in the concrete (photo taken on 9 September 2020).

This box was then repeatedly examined with imaging techniques—including the ScanVan of the Hamburg customs department and the pmCT of the Institute of Legal Medicine in Hamburg and stored on the roof of the Institute of Legal Medicine in between. Over the course of a year, the concrete block was scanned about every other week and a total of twenty-six times. The position of the pig’s head and paw inside the concrete is shown in Figure 2a,b. The first X-ray and pmCT images were taken on 14 September 2020 and the last scan on 25 August 2021. On 14 September 2021, the pig’s remains were chiseled out of the concrete with a hammer and chisel, examined again with X-ray and pmCT and macerated with tap water (approx. 40 °C) and enzyme-active soap (product name: Tergazyme-enzyme-active powdered detergent; Alconox, New York, NY, USA).

In addition to the radiological examinations, weather data were also collected, as the experiment was set up on the roof of the forensic institute from 23 September 2020, and was thus exposed to the ambient weather. These data were taken from a weather station in Hamburg Fuhlsbüttel, as this is the closest weather station to our institute (air distance: approx. 6 km) [24,25], and are available on reasonable request.

The X-ray device from the Hamburg customs department has a lower radiation density than conventional human X-ray devices. The colors depend on the density of the material being X-rayed. These colors can be used to draw conclusions regarding the scanned material. In general, organic materials are shown as orange, and inorganic material, for example metal and concrete, are blue. Furthermore, bones, plastic, aluminum, salts, etc., are shown in green, and anything black could not be penetrated by the radiation due to its high density. The pig’s head and paw appear blue because they are covered by the concrete and therefore appear inorganic due to the increased density of the layer above and below.

For a better comparison with similar studies, Galloway and Megyesi’s models were used for the decomposition stages once the remains were removed from the concrete [26,27]. Galloway et al. describe four stages of decomposition: fresh, early decomposition, advanced decomposition and skeletonization. Megyesi et al. adapted these stages and developed the Total Body Score (TBS), which is anatomically divided into head and neck, trunk, and limbs. In this case, both the head and neck and the limb tables were used to describe the changes visible in our pig. Megyesi et al. also added the use of accumulated degree days (ADD), which are calculated by adding the average daily temperature between the date of death and the date of detection. The ADD represent the accumulation of thermal energy needed for chemical and biological reactions during decomposition [27]. It is therefore mainly based on time and temperature, which measures the total amount of energy supplied to a system. Another factor influencing the decomposition rate is the carcass size and the accessibility for necrophagous insects. The cadaver size itself does not matter if the insects cannot gain access, which is the case for encased bodies [28].

The effectiveness of the so-called backscatter device was also assessed as part of this study. This is a portable X-ray device, which is used by Hamburg customs to search for drugs behind or inside obstacles such as doors or containers.

## 3. Results

The backscatter device could not penetrate deep enough into the concrete and therefore proved ineffective in this forensic setting.

In general, the outlines of the head and paw were clearly visible on both the X-ray and pmCT. On the pmCT, one could additionally see differences between air, osseous structures, and soft tissues, while on the ScanVan image, different compositions such as organic or non-organic material were made visible with different colors.

The projectile, located in the eye socket of the pig was easily distinguishable from the surrounding structures. Its position was stable over the years and clearly visible in every image. Even after the pig’s head was gouged out after 12 months, it was still in place.

**Figure 3:**
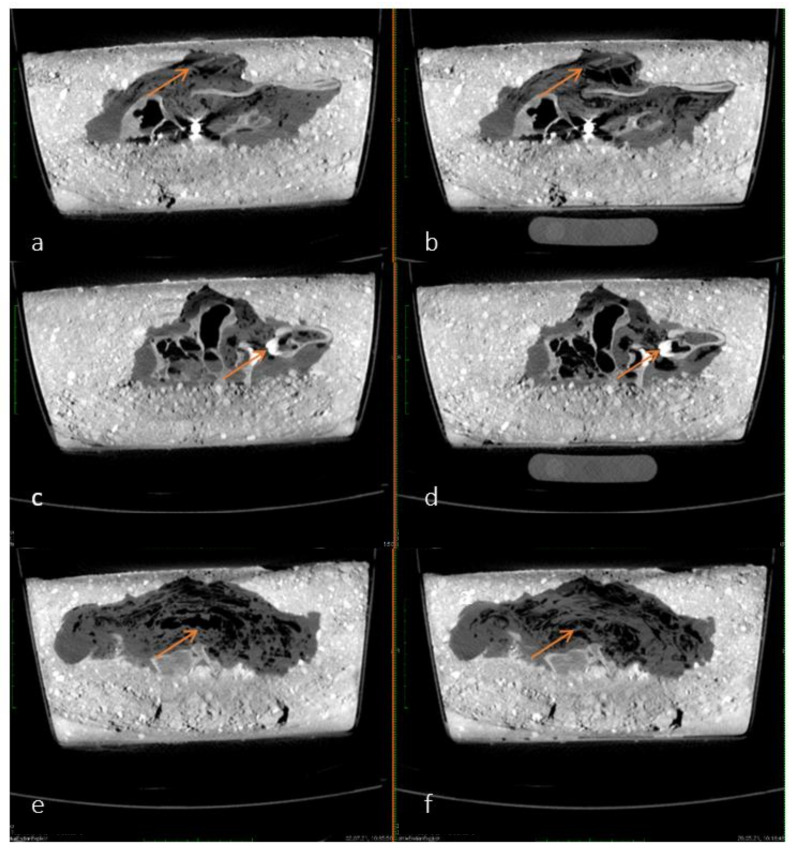
(**a**–**f**) Comparison of pmCT images in sagittal plane from 20 May 2021 (left—**a**,**c**,**e**) and 2 July 2021 (right—**b**,**d**,**f**). Examples of air accumulation and redistribution are shown by arrows.

As time progressed, the accumulation and continuous redistribution of air bubbles around the carcass became visible in different slices on the sagittal plane of the pmCT images. The air is exemplified as diffuse black areas marked with arrows in Figure 3a–f. The time interval shown in Figure 3—between 20 May and 2 July 2021, was chosen because the redistribution of air was most visible on these two differential images. These minimal changes occurred continuously in lesser amounts over the course of a year and could therefore not be associated with a specific point in time.

Over time, an odor was released from the concrete block, and, in addition, a hole was discovered on the enclosed head on 27 July 2021, which was probably caused by a bird.

**Figure 4:**
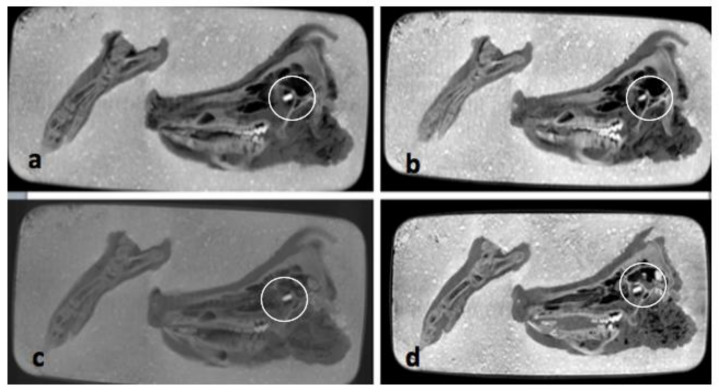
(**a**–**d**) Comparisons of pmCT images in axial plane from 30 September 2020 (**a**), 21 December 2020 (**b**), 3 March 2021 (**c**) and 25 August 2021 (**d**). The projectile is marked with a circle in all four images.

The pmCT scans in the axial plane (see Figure 4a–d), do not show any relevant changes over the course of one year. The bones and soft tissues are clearly visible on each scan, as is the projectile inside the orbit. The thickness and shape of soft tissues covering the bones remained almost unchanged over time. The amount of air inside the sinuses and soft tissues fluctuated, but there is no clear direction over time.

**Figure 5:**
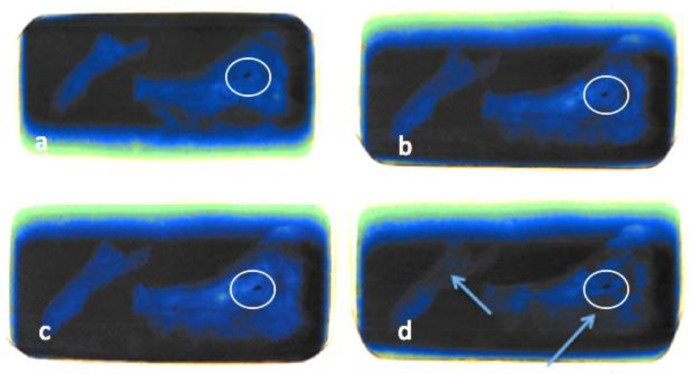
(**a**–**d**) Comparisons of X-ray images from 30 September 2020 (**a**), 21 December 2020 (**b**), 3 March 2021 (**c**) and 25 August 2021 (**d**). The darkening is shown by arrows. The projectile is marked with a circle in all four images. Quality of the images is due to the reduced radiation density in the portable X-ray machine.

On the exemplified radiographs in Figure 5a–d, a darkening can be seen covering the pig’s head and paw and progressing over time. This could be an indirect sign of decomposition of the soft tissues. The outline and the projectile in the eye socket of the pig are clearly visible in each image. The projectile can be seen in black due to the high combined density of the metal and the surrounding concrete.

**Figure 6:**
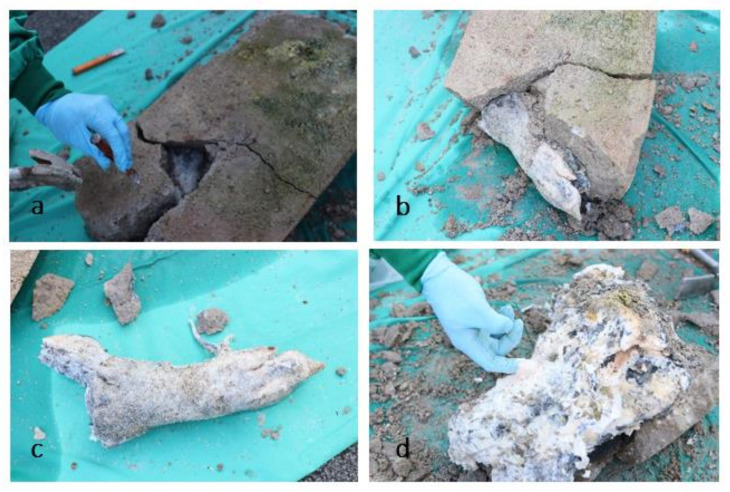
(**a**–**d**) Pictures taken on 14 September 2021 during chiseling out the carcass. (**a**) Shows the first phase of detasselling with the removal of the first large cement block. (**b**,**c**) Shows the remains of the paw. (**d**) Shows the advanced decomposition of the head.

On 14 September 2021, the concrete was gently removed (see Figure 6). First, the paw was revealed. The skin had a black-grey discoloration that appeared like a chemical burn of the outer layer, and parts of the skin remained stuck to the concrete. However, the skin, soft tissues, and bones of the paw were still intact, and only slightly altered by decomposition. These changes would fit within the period of early decomposition, with a score of 3 from the TBS table for limbs.

This score cannot be directly compared to a specific PMI under “normal” circumstances, as there are several factors that additionally influence the rate of decomposition, e.g., the sum of ADD, weather effects, etc.

The skin and soft tissues on the skull were completely decomposed and had a slimy consistency. They were easily separated from the bone. Therefore, the head would be at the level of ten points with a bone exposure of over 50 percent of the assessed area, including moist decomposition. This part of the body obviously was at a more advanced stage of decomposition than the paw. When the head was removed from the concrete, much soft tissue remained attached to the concrete in addition to the skin, which is why the condition of the skin could not be assessed further or in more detail. The condition of the paw corresponded to the condition previously seen on the X-ray and pmCT images. The head, on the other hand, supplied a completely different picture than would have been expected after seeing the images compared to its physical integrity. The discrepancy between the anatomical impression and its ‘virtual image’ can be explained by the properties of concrete, which formed an impression of the carcass and hardened around it, and therefore, did not lose its shape when the innards dissolved.

**Figure 7:**
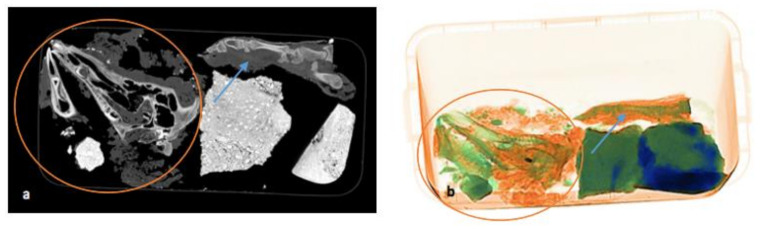
(**a**,**b**) Comparison of pmCT (**a**) and X-ray image (**b**) on 14 September 2021 during the last scan. The disarticulation of the bones and disintegration of soft tissue (red circle) of the head and intactness of the paw (blue arrow) can be seen.

On the final pmCT/ X-ray image in Figure 7a,b, the paw is still intact, with bones and soft tissues around it. The bones of the head are disarticulated due to the disintegration of the soft tissue. The projectile is still visible in the orbit.

## 4. Discussion

This empirical study was inspired by the aforementioned case, where the mobile X-ray device of the Hamburg customs department proved to be a particularly useful, ready-to use and fast investigation tool. As a result, it can be said that X-ray and pmCT are useful for finding and identifying human/mammalian remains even in difficult media such as concrete, and can thus help to reduce periprocedural damage. Furthermore, both X-ray and pmCT were able to detect decomposition changes with increasing time, but results could only show a significantly slowed rate of decomposition in concrete compared to putrefactive changes in an open environment and could not be used to assign specific changes to a specific point in time.

The total body score in combination with ADD, which are often used to assess the degree of decomposition, was not fully applicable in the present case, because normally the three scores of each table would be added, which would then result in the total body score. In this study, only two body parts were encased, insects were excluded from the decomposition process, and there was relative insulation in the concrete against temperature, humidity, and sunlight. However, the tables were still applied to the remaining body parts after the concrete had been removed, to supply some measures of comparison to similar other studies including encased carcasses.

In the studies by Martin et al. [29] and Gibelli et al. [30], the pigs were also encased in concrete and decomposition was measured at different times according to the TBS and the different stages of decomposition. As in our study, temperature, total precipitation, wind speed, and relative humidity were measured. Their results showed a significantly slowed decomposition of the encased carcasses with a TBS of 10–12 after one year.

In our study, the paw was still in the early stage of decomposition (TBS of limbs: 3), while the decomposition of the head had progressed more and was already in the advanced stage (TBS of head and neck: 10). This could be due to the higher humidity and more intense autolytic processes inside the head where there is more soft tissue and brain matter as well as air in the sinuses and pneumatized bones.

This TBS result differs from the other studies, as the TBS we added only for the head and limb without the trunk is thirteen points and therefore shows more advanced decomposition in our environment.

A similar finding to ours was the black discoloration of the outer skin layer and the separation of epidermis and dermis due to chemical ‘burning’ by the concrete [29]. This can be explained by the high proportion of calcium oxide in the concrete, which is largely basic and undergoes an exothermic reaction upon contact with water [31].

The different TBS results in the two aforementioned studies and our study, conducted in Hamburg, could be explained by the stronger influence of external factors due to different environmental conditions. Another explanation could be the different composition of the concrete or cracks and holes that developed over time [30]. In our study, odors were released from the concrete, indicating a lower insulation and lower density compared to other studies where no odors were released [11]. On 27 July 2021, a hole was additionally discovered on top of the encased head. As there was no permanent video observation of the set up, it can only be assumed that the hole was caused by a bird in combination with the expansion of air bubbles and the increasing porosity of the concrete. However, the hole did not lead to colonization by insects. The subject was not visible through the hole, but it can be surmised, that this led to a greater exposure to the environment. It can therefore be assumed, that the decomposition shown here was more susceptible to temperature, precipitation and humidity or even slight scavenging activity by birds than in other studies.

Our study was the first to investigate this specific set up, with encased pig parts in combination with continuous radiological imaging. It proved the value of such methods in specific forensic cases during the investigation process on site and in the Institute of Legal Medicine. Since, in many larger cities, it is possible to request mobile X-ray devices from Customs, it is advisable to make use of these devices to ensure the safe removal from concrete and thus preservation of the body and its possible clues.

X-ray and pmCT methods each showed added value and limitations. The advantages of the portable X-ray are that it can be used quickly in the field and can distinguish between varied materials such as organic and inorganic. However, it did not show as much detail as the pmCT and showed little evidence of a slow decomposition. PmCT images were better at distinguishing between air, bone, and soft tissue structures and better at suggesting decomposition changes due to the accumulation and redistribution of air. A limitation for pmCT, however, is a fixed location and thus, there is a lack of possibility or access to use the device on site. Both imaging techniques can only scan a limited range of objects in terms of weight and size and are therefore of limited value in cases where victims were encased as a complete body inside floors or walls. As a first step towards solving this problem, smaller, portable X-ray devices could be evaluated as next steps. The technical possibilities of such small devices are still limited and need to be thoroughly investigated in the future.

In addition, further studies should be encouraged in the future to compare encased and non-encased specimens also from other body parts and investigate a larger sample size. Adding radiological imaging to the list of examinations, a more frequent sampling or longer duration of the experiment could result in the development of a timeline of changes and a calculation of the extent of delay in decomposition in concrete.

A limitation of this study is the specific environmental setting, which makes the comparison of results only possible in similar weather conditions, regarding temperature, total precipitation, sun hours and humidity. Data on these details are available on reasonable request but were not included in full in this article to ensure its reasonable length/content ratio.

In the studies cited in the introduction, as well as in the mentioned case report, combined and sufficient autopsy and radiological investigations, among others, proved to be valuable for the identification, localization, and minimization of artefacts during excavation.

## 5. Conclusions

Radiological techniques such as X-ray and pmCT can be used to identify and localize body parts in concrete, helping to safely remove them from the substrate and thus reduce periprocedural damage. In contrast, assessing the state of decomposition and obtaining indications of the postmortem interval is hardly possible even with the inclusion of postmortem imaging. A minimal decay could be detected on both X-ray and pmCT, but it was not possible to assign a specific time point because these changes occurred continuously and in lesser amounts. X-ray and pmCT both have their value and limitations in such cases, including the possibility of detailed imaging of different structures in different planes on CT and the portability and use of the X-ray device in the field. The limitations of this include the stationary location of the pmCT and the less detailed image of the X-ray. In addition, the images did not reflect the actual situation of the head, which could be explained by the properties of the concrete, forming an impression around the carcass.

In summary, although radiological imaging modalities can be helpful in locating, identifying, and assessing encased and dismembered victims, such crime scenes still pose various difficulties and remain a challenge even for experts.

## Data Availability

Data are available on request due to restrictions. The data presented in this study are available on request from the corresponding author. The data are not publicly available due to necessary consultation with the Hamburg customs department.
